# A simple and effective method to purify and activate T cells for successful generation of chimeric antigen receptor T (CAR-T) cells from patients with high monocyte count

**DOI:** 10.1186/s12967-022-03833-6

**Published:** 2022-12-19

**Authors:** Haiying Wang, Shih-Ting Tsao, Mingyuan Gu, Chengbing Fu, Feng He, Xiu Li, Mian Zhang, Na Li, Hong-Ming Hu

**Affiliations:** 1 Department of Research and Development, Hrain Biotechnology Co., Ltd., 9th Floor, Building 1, 1238 Zhangjiang Road, Pudong New District, Shanghai, China; 2 Department of Manufacturing, Hrain Biotechnology Co., Ltd., 9th Floor, Building 1, 1238 Zhangjiang Road, Pudong New District, Shanghai, China

**Keywords:** Chimeric antigen receptor T (CAR-T) cells, T-cell purification, T-cell activation, Monocyte, Design of experiments (DoE)

## Abstract

**Background:**

Chimeric antigen receptor T (CAR-T) cells are genetically modified T cells with redirected specificity and potent T-cell-mediated cytotoxicity toward malignant cells. Despite several CAR-T products being approved and commercialized in the USA, Europe, and China, CAR-T products still require additional optimization to ensure reproducible and cost-effective manufacture. Here, we investigated the critical parameters in the CD3^+^ T-cell isolation process that significantly impacted CAR-T manufacturing's success.

**Methods:**

CAR-T cells were prepared from cryopreserved peripheral blood mononuclear cells (PBMC). The thawed PBMC was rested overnight before the CD3^+^ T cell isolation process using CTS^™^ Dynabeads^™^ CD3/CD28. Different isolation media, cell-bead co-incubation time, and cell density were examined in this study. Activated CD3^+^ T cells were transduced with a gamma retroviral vector carrying the CD19 or BCMA CAR sequence. The CAR-T cells proliferated in a culture medium supplemented with interleukin 2 (IL-2).

**Results:**

CD14^+^ monocytes hindered T-cell isolation when X-VIVO 15 basic medium was used as the selection buffer. The activation of T cells was blocked because monocytes actively engulfed CD3/28 beads. In contrast, when DPBS was the selection medium, the T-cell isolation and activation were no longer blocked, even in patients whose PBMC contained abnormally high CD14^+^ monocytes and a low level of CD3^+^ T cells.

**Conclusions:**

In this study, we discovered that selecting CD3^+^ T-cell isolation media is critical for improving T-cell activation, transduction, and CAR-T proliferation. Using DPBS as a CD3^+^ T cell isolation buffer significantly improved the success rate and shortened the duration of CAR-T production. The optimized process has been successfully applied in our ongoing clinical trials.

*Trial registration* NCT03798509: Human CD19 Targeted T Cells Injection Therapy for Relapsed and Refractory CD19-positive Leukemia. Date of registration: January 10, 2019. NCT03720457: Human CD19 Targeted T Cells Injection (CD19 CAR-T) Therapy for Relapsed and Refractory CD19-positive Lymphoma. Date of registration: October 25, 2018. NCT04003168: Human BCMA Targeted T Cells Injection Therapy for BCMA-positive Relapsed/Refractory Multiple Myeloma. Date of registration: July 1, 2019

**Supplementary Information:**

The online version contains supplementary material available at 10.1186/s12967-022-03833-6.

## Background

Chimeric antigen receptor T (CAR-T) cell therapy is a profound breakthrough that significantly changed the medical practice for hematological malignancies in the twenty-first century. The long-lasting efficacy in hematological malignancies, including acute lymphoblastic leukemia (ALL) and B-cell non-Hodgkin’s lymphoma (NHL), offers the cure for many patients [1–3]. Nevertheless, a broad application of CAR-T therapy in clinics is still faced with many challenges; for example, the toxic side effect due to cytokine release syndrome (CRS) and immune effector cell-associated neurotoxicity syndrome (ICANS), the rapid recurrence of cancer due to the exhaustion and short-term persistence of CAR-T cells after infusion, and minimal efficacy on solid tumors [4, 5]. Most studies focus on improving CAR structure design to maximize effectiveness and minimize side effects. Besides the scientific and clinical problems that need to be resolved, many manufacturing and engineering issues require focused attention to have a CAR-T cure available to more patients. Unfortunately, the problems we face during the manufacturing CAR-T cells are less discussed, and a standard manufacturing process for CAR-T cells has not yet been established. In March 2022, U.S. Food and Drug Administration (FDA) published draft guidance providing considerations for the development of CAR-T products [6]; we believe this new guidance set a pathway for us to follow on how to optimize and standardize the CAR-T cell manufacture process.

Unlike many traditional pharmaceutical products, autologous CAR-T products are living products manufactured from whole blood or leukapheresis collected from each patient as the starting material. Generally, the manufacture of CAR-T cells consists of five principal processes. The first step is the processing of starting material. The leukapheresis or whole blood goes through either peripheral blood mononuclear cells (PBMC) separation by density gradient centrifugation and wash procedure to remove red blood cells. The second step is the T-cell enrichment step, typically achieved using paramagnetic beads by either a positive or negative selection method. Beads conjugated with anti-CD3 with and without anti-CD28 antibodies could be used for positive selection and T-cell activation in one step. Alternatively, PBMCs were first depleted of non-T cells using paramagnetic beads conjugate with various antibodies targeting CD19/CD14/CD16/CD56 to deplete B cells, monocytes, and NK cells T-cell activation reagents were added later to promote T cells viral transduction and proliferation. Third, T cells are transduced with CAR genes targeting specific antigens, such as CD19 or B-cell maturation antigen (BCMA), via a viral vector or non-viral techniques. Fourth, transduced CAR-T cells proliferate and expand in media supplemented with recombinant interleukin 2 (IL-2) and other cytokines.

Finally, expanded CAR-T cells are cryopreserved and kept in liquid nitrogen before infusion [7–10]. Despite the overall process looking very similar, the detail in each process step can differ significantly among different labs or companies, and between 7 and 9% of manufacturing failure rates were reported in clinical trials of Kymriah (tisagenlecleucel) and Tecartus (brexucabtagene auto excel) [1, 11, 12]. The failure to generate CAR-T products may result from poor quality of patients’ PBMC or sub-optimal manufacturing processes [5, 10]. The critical questions are whether we can and how to improve the rate of success even if the quality of patients’ PBMC is suboptimal. The suboptimal quality of PBMC is a crucial issue because most patients will have no second chance if their products fail.

The essential step in CAR-T manufacture is the purification and activation of CD3^+^ T cells. PBMC, isolated from leukapheresis and the expected starting material for CAR-T production, consists of normal lymphocytes, monocytes, leukemia, and lymphoma cells. Therefore, the T-cell purification step is necessary for successful CAR gene transduction and subsequent T-cell expansion, but also it is crucial for safety considerations. This process is usually achieved by incubating PBMC with anti-CD3/CD28 antibody-coated paramagnetic beads or degradable nano matrix in a suitable isolation buffer, followed by a capture step to positively select CD3^+^ T cells with a magnum [8, 10]. Previously, we used Dynabeads™ Human T-Activator CD3/CD28 for T-cell isolation and activation. However, we noticed that in some patients’ PBMC was particularly difficult to generate enough CAR transgene positive T cells when the T-cell count in PBMC was lower than usual; thus, many patients were excluded from our clinical trials. In this study, during the investigation of the culprit for the manufacturing failure, we unexpectedly observed that monocyte-rich PBMC samples experienced poor activation and expansion of T cells. By retrospectively examining batch records from our CAR-T clinical trials, we found a link between the types of buffers used in the CD3^+^ cell isolation step and the degree of CAR-T cell expansion. Subsequently, we found that buffer types are the key input influencing CAR-T cell expansion response via establishing the “Design of experiments” (DoE) to evaluate factors of the CD3 isolation process, including buffer types and other process parameters. The optimized process based on this DoE analysis was applied to our ongoing clinical trials and greatly improved CAR-T cell expansion in monocyte-rich PBMC samples. The modification presented in this study could be easily implemented in an already established and widely-used protocol to improve CAR-T products’ quality and success rate significantly.

## Results

### ***The percentage of CD14***^+^***monocytes negatively affects the efficiency of bead-based T-cell selection and activation***

**Fig. 1:**
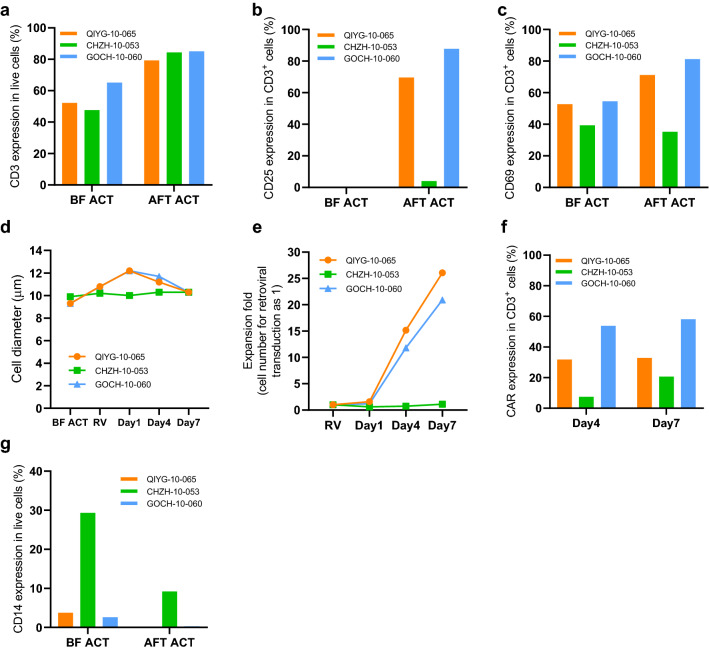
CAR-T cell characteristics when using CAR-T culturing medium as the T cell selection buffer. This figure showed the outcome of CAR-T cell manufacture when CAR-T culturing medium was used as the T cell bead-based selection buffer. **a** The percentage of CD3 positive cells in PBMC before positive selection with anti-CD3/28 Dynabeads (BF) and positively selected cells after 44–52-h activation (AF). **b** The percentage of CD3^+^ cell population that express surface CD25 marker (**b**) or CD69 (**c**) before and after activation. **d** Cell diameter measurement results throughout the CAR-T production process. RV, the day of retroviral transduction and just before cell selection (AF). Day 1–7 indicates days after retroviral transduction. **e** Fold expansion of CAR-T cells after retroviral transduction. **f** Percentage of CD3^+^ cells express CAR at days 4 and 7 after retroviral transduction. **g** The percentage of live cells that were positive for CD14 surface marker before selection and after activation

Previously, we encountered a small percentage but still significant CAR-T manufacture failure in our investigator-initiated trials (IIT) or registered clinical trials (NCT03798509, NCT03720457, and NCT04003168). We reasoned that more patients would be treated if we could improve the manufacturing process to reduce manufacturing failure. At the beginning of this study, we encountered a problem during the first step of CAR-T manufacture, e.g., the bead-based CD3^+^ T-cell isolation step. Our original process, which strictly followed the manufacturer’s procedure, used Dulbecco's phosphate-buffered saline (DPBS) or X-VIVO15 basic medium as the isolation buffer depending on the CD3^+^ cell percentage in PBMC [13]. DPBS was used for PBMC with the CD3^+^% higher than or equal to 30%, whereas we used X-VIVO 15 basic medium if the CD3^+^% was below 30%. To simplify the process, we tested the possibility of using a CAR-T culturing medium as the isolation buffer regardless what the percentage of CD3^+^ T cells in the purified PBMC. Three batches of PBMC samples from patients’ apheresis were randomly chosen as starting material for this experiment. Patient CHZH-10-053 was diagnosed with NHL, while the other two were diagnosed with multiple myeloma (MM). Although the efficiency of CD3^+^ cell selection was similar when different donor PBMCs were used, we observed that T cells isolated from one donor PBMC was poorly activated (Fig. 1a). About 48 h after selection and activation, the expression of activation markers CD25 and CD69 on T cells was normal for two patients (GOCH-10-060 and QIYG-10-065). Still, their expression was minimally induced for the patient (CHZH-10-053) (Fig. 1b and c). CD25 expression on T cells from CHZH-10-053 was below 10%, and CD69 expression was reduced after selection. In addition, the diameter of activated T cells from CHZH-10-053 was not increased, and there was no CAR-T proliferation following culture in IL-2-containing media (Fig. 1d and e).

Furthermore, little CAR expression on CD3^+^ T cells could be detected, which was consistent with the failure of T-cell activation (Fig. 1f). After a careful flow cytometry analysis of different subsets of immune cells present in the PBMC before and after T-cell selection, we found that the PBMC of CHZH-10-053 contained an abnormally high percentage of CD14^+^ cells before or after T-cell selection. One-third of the PBMC were CD14^+^ cells before selection, and still, 10% of PBMC were CD14^+^ after selection (Fig. 1g). The results obtained from one patient suggested that CAR-T cell proliferation and CAR transduction were negatively impacted by the abundance of CD14^+^ monocytes in the PMBC

### ***The choice of T-cell selection buffer rather than the percentage of CD14***^+^***%CAR-T greatly affected the T-cell proliferation and CAR-T manufacture duration***

**Fig. 2:**
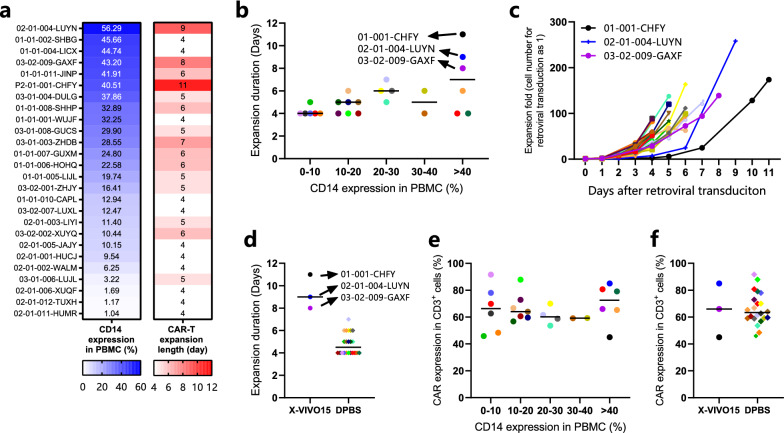
CAR-T manufacturing data of patients participating in phase I clinical trials. The figure shows the PBMC composition, CAR expression, and CAR-T cell expansion data of 26 patients who participated in CD19 and BCMA CAR-T phase I clinical trials. **a** Heatmap analysis of CD14 percentage in patients’ PBMC and CAR-T expansion days required to reach the requested dosage. The left column shows the CD14 percentage (1.04–56.29%) in PBMC from high to low before cryopreservation, and the right shows CAR-T expansion days (4–11) of the corresponding patient. **b** CAR-T cell expansion duration of each patient was plotted against CD14 expression levels in PBMC. Among all patients, CAR-T cells (01-001-CHFY, 02-01-004-LUYN, and 03-002-009-GAXF) took the longest to expand. Expansion duration: the days between the completion of retroviral transduction and the filling of the CAR-T product. **c** Individual expansion rate of CAR-T cells of all patients. The expansion curve of T cells from patients (01-001-CHFY, 02-01-004-LUYN, and 03-002-009-GAXF) are marked with black, blue, and purple, respectively. **d** CAR-T cell expansion duration divided by different CD3^+^ T cell isolation buffers. **e** CAR expression percentage in CD3^+^ cells of each patient was plotted against CD14 expression levels in PBMC. **f** CAR expression percentage in CD3^+^ cells was compared between groups of CAR-T cells selected with two isolation buffers

The striking observation from one patient prompted us to perform a retrospective analysis of results from our phase I CAR-T clinical trials in which 25 patients’ manufacturing batch records were available. First, we created a heatmap to show the level of % CD14^+^ cells in a patient’s PBMC from high to low, along with the number of days required for successful product filling for each patient after retroviral transduction (5 × 10^5^–2 × 10^6^ CAR^+^ cells per kg weight in CD19 CAR-T clinical trial and 3 × 10^6^–9 × 10^6^ CAR^+^ cells per kg weight in BCMA CAR-T clinical trial) (Fig. 2a). CD14^+^ monocytes (1.04–56.29%, median at 19.74%) were significantly higher than healthy donors (around 5%) [14]. The heatmap clearly showed that the number of days, e.g., the duration of production, was much longer when the levels of the CD14^+^% in PBMC were higher. By dividing patients into two groups using 40% CD14^+^% in PBMC as the cut-off (Fig. 2b), we found that CAR-T cell expansion duration for three such patients was much longer than for others (as indicated with arrows and patient IDs in Fig. 2b).

Other than the CD14^+^% monocytes, the duration of expansion of our phase I trials could also be affected by the low CD3^+^% level in PBMC, which results in fewer cells used for retroviral transduction and expansion. Furthermore, the higher CAR-T cell number dependent on patients’ weights and dose levels stipulated in clinical trials could also affect manufacturing duration. Therefore, we plotted the fold of T-cell expansion against the production duration time. The results partially supported our hypothesis that the abnormally high CD14^+^% in PBMC negatively impacted the CAR-T cell proliferation in three of six patients (Fig. 2c). However, we also noticed that CAR-T cells from another three patients with abnormally high CD14% completed their expansion within the normal range of 4–6 days. This observation suggested that the level of CD14^+^% was not the only determining factor that led to the lower expansion rate of transduced T cells and prolonged expansion duration.

Further analysis of the difference in the manufacturing process used for these six patients showed that the T-cell selection buffer differed. As shown in Fig. 2d, the group's median expansion duration was nine days using X-VIVO15 basic medium (indicated with arrows) as an isolation buffer. In contrast, the expansion duration was cut in half (an average of 4.5 days) when DPBS was used. Unlike what we observed from one patient (CHZH-10-053) showed earlier, the CAR expression on the day of product filling was not affected by CD14^+^% in PBMC (Fig. 2e, f). Surprisingly, we also found a comparable CAR expression level (64% and 66%), whether DPBS or X-VIVO basic medium was used as the selection media. This surprising finding indicated that the isolation buffer was the critical factor, rather than the CD14^+^% in PBMC, that was the key determinant for CAR-T cell proliferation. Based on these results obtained from the lab and clinical trials, we concluded that X-VIVO15 basic medium as isolation buffer in the CD3^+^ T cell selection process could lead to defective T cell activation and CAR-T proliferation, mainly when CD14^+^% level in the patient’s PBMC was abnormally high. DPBS could be used for the T-cell isolation buffer no matter the CD14^+^% in PBMC.

### ***Adherent CD14***^+^***cells engulfing Dynabeads were observed during the T-cell selection process when using X-VIVO15 basic medium as a selection buffer***

**Fig. 3:**
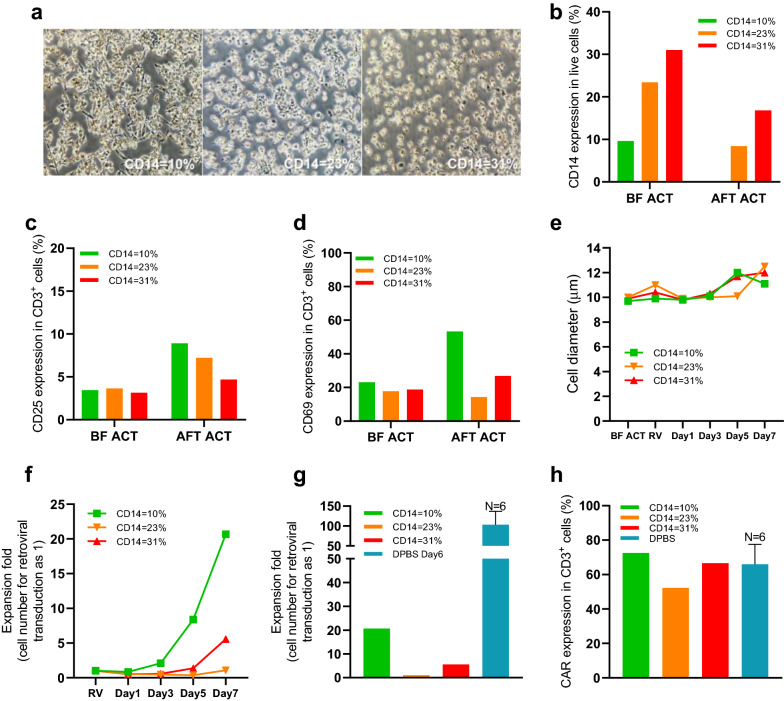
Manufacturing CAR-T cells using X-VIVO15 basic medium as T cell selection buffer. Using X-VIVO15 basic medium as the CD3^+^ T cell isolation buffer hampered the CAR-T cells manufacturing, and expansion deficiency was related to CD14^+^ cells. **a** Microscopic images showed numerous adherent cells with engulfed magnetic beads after 48-h T-cell activation inside the cell culture bag. **b** CD14 expression in live cells before CD3^+^ T cell selection and after 48-h T cell activation. The percentage of CD3^+^ cells express surface CD25 (**c**) or CD69 (**d**) before CD3^+^ T cell selection or after 48-h T cell activation. **e** Fluctuations of cell diameter values throughout the CAR-T manufacturing process. **f** Expansion fold of CAR-T cells after retroviral transduction. **g** This experiment compares the expansion fold on day seven after retroviral transduction and the average expansion fold on day 6 in the phase I clinical trial (6 patients with day six cell-counting data recorded). **h** CAR expression percentage in CD3^+^ cells at the end of CAR-T manufacture (the day of product filling) between this experiment and the phase I clinical trial (all 26 patients)

PBMC with different levels of CD14^+^ cells (10%, 23%, and 31%) were purposely selected and used to examine how the success rate of CAR-T manufacture would be affected by the content of monocytes. As done previously, thawed PBMCs were incubated with CD3/28 Dynabeads in an X-VIVO15 basic medium. T cells were isolated by positive selection and put into T-75 tissue culture flasks for additional incubation. After carefully examining flasks under an inverted microscope, we noticed many adherent cells on the culture surface, and cells were actively engulfing Dynabeads (Fig. 3a). Flow cytometry analysis of PBMC before and after cell selection found that a variable number of CD14^+^ cells persisted (8 to 17%) in the positively selected PBMC from two of the three donors whose CD14^+^ cells before the selection was relatively higher (Fig. 3b).

Both CD25 and CD69 expression were barely increased after activation, and T-cell blast formation was delayed until 5 or 7 days after retroviral transduction (Fig. 3c–e). Although the CAR-T expansion fold was higher in the low CD14^+^% group, the overall CAR-T expansion fold on day 7 was much lower than clinical trial data on Day 6 when DPBS was used as the selection buffer (Fig. 3f, g). Again, the CAR expression at the end of the culture was not affected by selecting an isolation buffer or CD14^+^% in PBMC. We found that the engulfment of Dynabeads by an excessive number of CD14^+^ cells could lead to the insufficient selection and ineffective activation. In one patient, the CD14^+^% cells in PBMC were close to normal (10%), but the T-cell expansion was still less than usual. We also found that positively selected T cells were still contaminated with 0.5% CD14^+^ cells. We suspected that these minor populations of CD14^+^ cells were still significant enough to consume Dynabeads and resulted in poor T-cell stimulation. However, we could not exclude the possibility that these CD14^+^ monocytes were immunosuppressive MDSC found in cancer patients that could actively suppress the T-cell activation [15]. These observations strengthened our speculation that using the X-VIVO15 basic medium as an isolation buffer could result in defective T cell activation and CAR-T expansion regardless of the CD14^+^% in the starting material.

### The DoE confirms the advantage and importance of DPBS as the selection buffer

To systematically optimize parameters that determine the optimal condition for CAR-T manufacture, we used DoE further to define an optimal T-cell selection and activation parameter. In addition to the choice of selection buffer, other parameters such as T-cell density during Dynabeads incubation and incubation duration were also included. In DoE, the process parameters we wanted to optimize were “input factors,” while the results we obtained were “output responses.” To create a design space or determine a better option for process parameters, we set up different “levels” of an input factor at the beginning of DoE. Input factors of our DoE in the T-cell isolation step are choice of isolation media (2 levels, DPBS or X-VIVO), T-cell density during Dynabeads incubation (3 levels, 4, 7, or 10 × 10^6^ cells/mL), and incubation duration (3 levels, 30, 45, 60 min). We focus on the output responses CAR-T expansion fold at the experiment endpoint and upregulation of T-cell activation markers after T-cell selection and activation, including CD25 expression and CD69 expression in T cells and cell diameter. Experiment groups in the DoE and the levels of factors investigated are listed in Table 1. PBMC with different levels of CD3^+^ cells (three samples for each of the following levels of CD3^+^% cells: < 30%, 30–60%, > 60%) were used in this DoE to ensure the complete application of this optimized process in the future clinical trials where a wide range of CD3^+^% in PBMC will be encountered. The DoE was repeated three times, and nine PBMC samples were used.

**Table 1:** Groups, factors, and factor levels of DoE

Group	CD3 + cells in PBMC (%)	Duration of incubation (min)	CD3 + cell density for incubation (e6 cells/mL)	Incubation buffer
#01	< 30	30	4	X-VIVO15
#02	< 30	30	10	DPBS
#03	< 30	60	4	DPBS
#04	< 30	60	10	X-VIVO15
#05	30–60	45	7	DPBS
#06	30–60	45	7	X-VIVO15
#07	> 60	30	4	DPBS
#08	> 60	30	10	X-VIVO15
#09	> 60	60	4	X-VIVO15
#10	> 60	60	10	DPBS

Factors and factor levels of each group in DoE are listed in the table

**Fig. 4:**
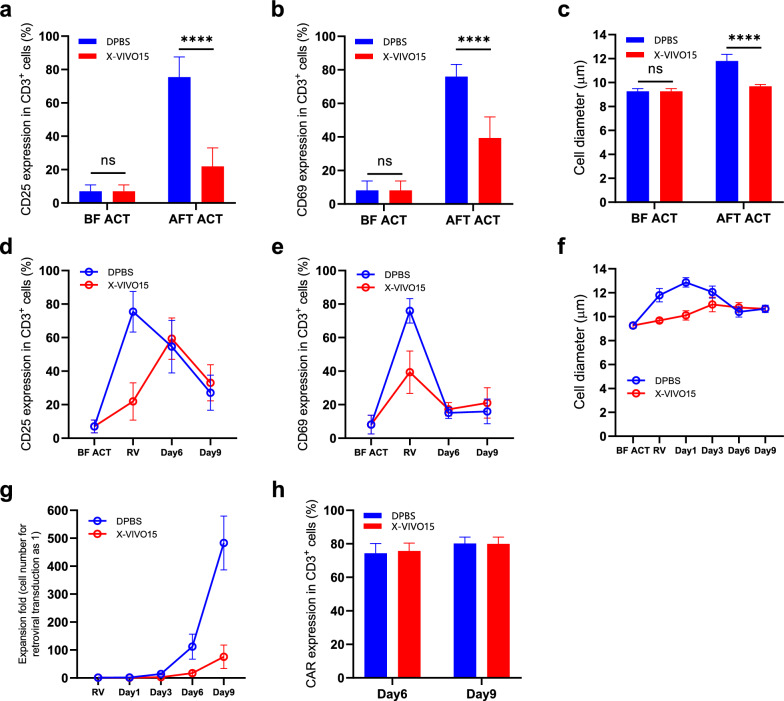
Significant differences were observed in CAR-T manufacture using different T cell selection buffers. This figure compares DPBS or X-VIVO15 basic medium as the CAR-T manufacturing process’s CD3 + T cell selection buffer. The DoE was repeated three times, and three PBMC samples were used in each repeat. Groups and factor levels in the DoE are shown in Table 1. **a** and **b** show the percentage of CD3^+^ cells that express CD25 (**a**) or CD69 (**b**) surface markers. In **c**, the cell diameter was measured before CD3^+^ T cell selection and after 48-h T-cell activation. In **a**, **b**, and **c**, an unpaired t-test was used, and a two-tailed P value was calculated; ns, P > 0.9999; ****, P < 0.0001. **d** Changes in CD25 expression percentage in CD3^+^ cells, **e** CD69 expression percentage in CD3^+^ cells, and **f** live-cell diameter in the CAR-T manufacturing process. **g** Expansion fold of CAR-T cells after retroviral transduction. **h** CAR expression percentage in CD3^+^ cells at days six and nine after retroviral transduction

By comparing data obtained from groups using DPBS and X-VIVO 15 basic medium as the T-cell selection buffer, we again observed the dramatic difference in the T-cell activation and CAR-T proliferation when different selection media were used. We found that T cells expressed higher levels of CD25 and CD69 and exhibited larger cell diameter at the 48-h activation point when DPBS was used as the selection medium compared to the X-VIVO 15 basic medium (Fig. 4a–c). In addition, we could see delayed and attenuated peak values of these activation markers throughout the CAR-T manufacturing process when the X-VIVO medium was used (Fig. 4d–f). At the end of the culture, the average CAR-T cell expansion fold of the DPBS group vs. X-VIVO15 was 483.1 and 75.8, respectively (Fig. 4g). CAR expression levels did not differ with different isolation buffers, and the average percentage of expression in CD3^+^ cells was over 70% at various time points after transduction (Fig. 4h). The other input factors in this DoE, namely T-cell density during Dynabeads incubation and incubation duration, did not reach statistical significance when three different levels were investigated and analyzed (see Additional files 1 and 2).

**Fig. 5:**
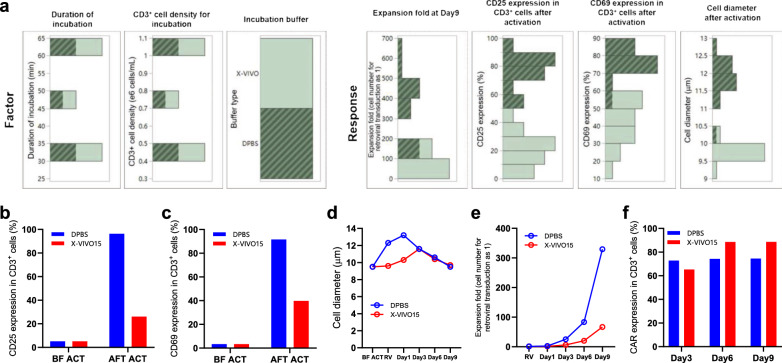
DoE analysis of T cell isolation parameters and the validation through CAR-T manufacture. This figure shows the results of DoE analysis for the three above parameters and the validation of the optimized T cell selection process with a new round of CAR-T manufacture. **a** DoE distribution analysis of input factors and output responses. The diagonal pattern shows the desired responses and their distribution in research factors. CD25 (**b**) and CD69 (**c**) expression percentage in CD3^+^ cells before CD3^+^ T cell selection and after 48-h T cell activation, live cell diameter changes (**d**), expansion fold of CAR-T cells after retroviral transduction (**e**), and CAR expression percentage in CD3^+^ cells at day 3, 6, 9 after retroviral transduction of CAR-T manufacturing process for DoE validation (**f**)

We used JMP software to generate a distribution report to visualize the relationship between input factors and output responses and to determine which levels of any process parameters were the most influential ones leading to desired outcomes. When we selected any one of the levels of input factors, this selection worked as a filter, and the output responses related to the level were highlighted with the same color/pattern as the selected group. Shown in Fig. 5a is the distribution report generated by JMP based on our DoE data. When we chose DPBS, one of the levels of the choice of isolation medium, the relative output responses were also highlighted with a diagonal pattern. The highlighted output responses include the higher CAR-T expansion fold, CD25 and CD69 expression on T cells after activation, and cell diameter. The distribution report indicated that using DPBS as an isolation medium is highly related to better T-cell activation and more significant CAR-T proliferation.

To experimentally validate the DoE outcome, we split one PBMC sample into two portions, using parameters determined in the DoE and the other using X-VIVO15 basic medium as an isolation buffer in CAR-T manufacture. The result perfectly validated the new process parameters specified by DoE, which resulted in sound activation, proliferation, and regular CAR expression (Fig. 5b–f). Parameters defined by DoE are shown in Table 2.

**Table 2:** Optimal choice or range of process parameters in T cell isolation process defined by DoE

Process parameter	Optimal choice or range
Isolation medium	DPBS
T-cell density during Dynabeads incubation	4*10^6^–10*10^6^ CD3^+^ cells/mL
Dynabeads incubation duration	30–60 min

Process parameters studied through DoE and their defined values are shown in the table

### ***The use of DPBS as an isolation buffer improved the success of CAR-T manufacture from PBMC with abnormally high-level CD14***^+^***cells and low-level CD3***^+^***cells***

**Fig. 6:**
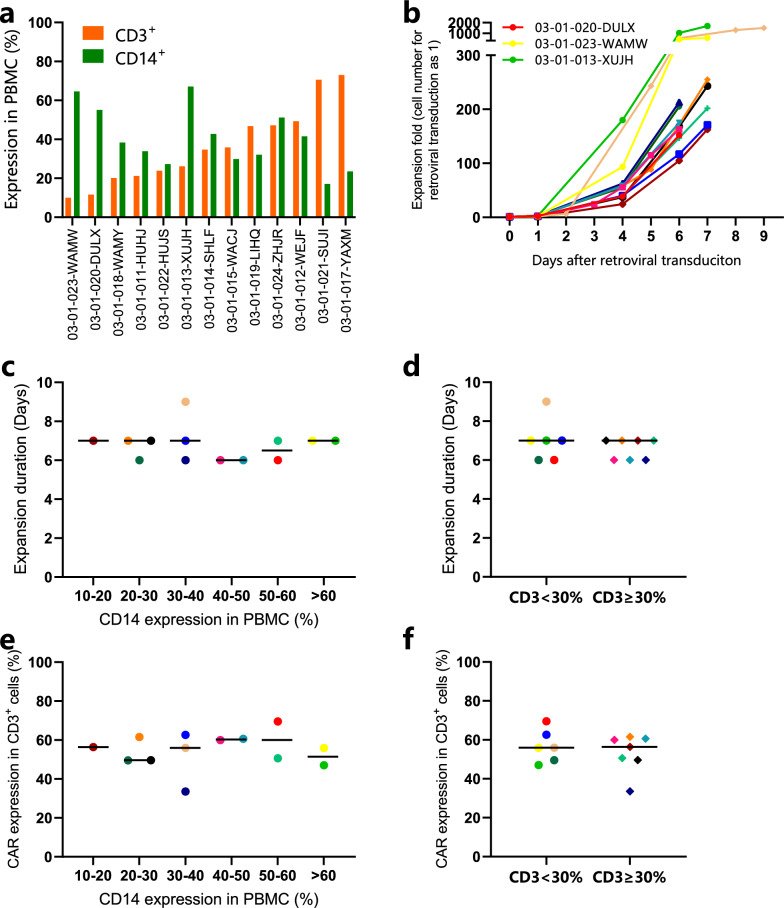
Results of BCMA CAR-T manufacture with optimized T cell selection process in the clinical trial. This figure shows that DPBS was successfully used as the T cell isolation buffer to manufacture anti-BCMA CAR-T cells for 13 patients in phase I clinical trial. **a** CD14 expression and CD3 expression in patients’ PBMC. **b** CAR-T cell expansion rate after retroviral transduction. The three patients with excessively low CD3^+^% in their PBMC are marked red, yellow, and green. CAR-T cell expansion duration of each patient was plotted against the percentage of CD14^+^ cells (**c**) or CD3^+^ cells (**d**) in PBMC before selection. CAR expression percentage in CD3^+^ cells of each patient plotted against the percentage of CD14^+^ (**e**) or CD3^+^ (**f**) cells in their PBMC

Because of the apparent improvement with DPBS as the T-cell selection buffer over our original process, we implemented the change to our manufacturing process. We produced CAR-T cells to treat 13 additional MM patients in an ongoing phase I BCMA CAR-T clinical trial (NCT04003168). A wide range of CD14^+^ and CD3^+^ cells were found in the PBMC of these patients (Fig. 6a). Analysis of CAR-T expansion fold showed more than 100-fold expansion for all 13 patients, even for patients with an abnormally high percentage of CD14^+^ cells in their PBMC (Fig. 6b). So far, we have a 100% success rate of CAR-T manufacture for 13 patients. A uniformity of expansion duration and CAR expression levels was observed regardless of CD14^+^ % or CD3^+^ % of PBMC in the starting materials (Fig. 6c, d, e). Noticeably, we have successfully generated CAR-T products from three patients we have failed to manufacture if the X-VIVO 15 medium was used as the T-cell selection medium (Fig. 6f). CAR-T products were successfully manufactured with this modified process for three patients whose PBMC had a very high CD14^+^ % and very low CD3^+^ % in their PBMC (03-01-020-DULX: CD14^+^ = 55.1%, CD3^+^ = 11.6%; 03-01-023-WAMW: CD14^+^ = 64.6%, CD3^+^ = 10.0%; 03-01-013-XUJH: CD14^+^ = 67.1%, CD3^+^ = 26.1%). This simple solution to use DPBS instead of X-VIVO culture medium in T-cell selection will significantly impact the CAR-T manufacturing process development and is currently adapted into several CAR-T product lines for our ongoing phase I/II clinical trials.

## Discussion

Adopting T cell therapies for cancer using genetically modified T cells started in the 1980s, and only recently, CAR-T-cell immunotherapy has experienced rapid development and clinical success [16]. From 1997 to 2020, more than 700 CAR-T-related clinical trials were registered on ClinicalTrials.gov [17]. Most importantly, eight CAR-T products in global markets were already approved to treat patients with leukemia, lymphoma, or MM [18]. These current and future products differ significantly in the choice of targeting antigens and scFv, the design of constructs of CAR molecule, the clinical trial design, and other factors that determine the efficacy, safety, and success of CAR-T products. Optimization of the CAR-T-cell manufacturing process is equally critical for CAR-T therapy's success.

The first challenge we face in CAR-T manufacture is how to enrich and activate T cells from a patient’s PBMC since a proper activation of T cells is critical for efficient CAR gene transduction and proliferation of transduced T cells. The Dynabeads™ Human T-Activator CD3/CD28, superparamagnetic micron beads coated with anti-CD3 and anti-CD28 mAbs are widely used for research, clinical trial, or commercial CAR-T products. The first FDA-approved CAR-T product, Kymriah, used Dynabeads [19]. T-Cell TransAct™, a nano matrix covalently bounded with anti-CD3 and anti-CD28 agonists, served as a T-cell activator and was similarly developed by Miltenyi Biotec [20]. Expamer set by IBA is another activation reagent soluble in anti-CD3/CD28 Fab fragments linked to a recombinant Streptactin backbone [21]. Although manufacturers offer recommended procedures and general guidance on using their products, they can only serve as the guideline and starting point for more in-depth, individualized process development. Our current study unveiled that the buffer choice for the T-cell selection is decisive for CAR-T manufacture if Dynabead from ThermoFisher is used.

First, when using a medium containing human plasma as the isolation buffer in the Dynabeads co-incubation step, T cells from some donors were poorly activated, and their CAR-T cells barely proliferated. To determine the underlining reason, we did a retrospective analysis of data from our clinical trial. The original process development strictly followed the recommended choice of T-cell selection buffer based on the CD3^+^% found in PBMC. We discovered that failed activation and low proliferation of T cells happened more often when PBMC contained a higher percentage of CD14^+^ monocytes. Initially, we suspected that the high CD14^+^% in PBMC was the potential culprit for the failure. Several publications in the literature showed that CD14^+^ monocytes in mobilized peripheral stem cells (PSC) and PBMC isolated from cancer patients inhibited T-cell activation. In the 1990s, scientists realized that CD14^+^ monocytes in granulocyte–macrophage colony-stimulating factor (GM-CSF) mobilized PSC products, and peripheral blood post-transplantation could induce apoptosis of activated T cells [22, 23]. As CAR-T therapy gained wider popularity, researchers also noticed that monocyte posted a negative influence on CAR-T manufacture. A higher proportion of myeloid cells in PBMC or PBSC have been reported with poor or failed T-cell expansion field [24, 25].

In 2021, two groups presented data conveying the importance of CD14^+^ cell depletion in the CAR-T manufacturing [26, 27]. Wang and colleagues reported that depletion of monocytes from the starting material benefits the CAR T cell activation, transduction efficiency, and tumor-killing ability and could also improve the T cell stemness, which might promote the in vivo expansion and persistence of infused CAR-T products [26]. Their study set a 40% CD14^+^ monocyte content threshold for an additional plastic adhesion step needed to remove adherent monocytes for an overall successful manufacturing rate of 97%. Noaks et al*.* presented similar results: CAR-T cells made from CD14^+^ cells-depleted samples outperformed untouched leukapheresis in various aspects, including transduction efficiency, cytotoxicity, and T cell stemness [27]. Studies by Stroncek et al*.* a few years ago also suggested that plastic adhesion helps remove CD14^+^ cells and benefit the CAR-T cell expansion [24, 28].

Next, we planned experiments with the DoE tool to optimize the critical parameters. Surprisingly, we found that the choice of isolation buffer between X-VIVO15 and DPBS was the critical feature that determined the success of T-cell activation and CAR-T cell expansion. By using DPBS as the isolation buffer, we significantly improved the T-cell activation and proliferation of CAR-T cells as compared with cell culture media used in our original process, even for patients whose PBMC contained more than 60% CD14^+^ monocyte and from whom we failed to generate enough CAR-T cells to treat patients. One major characteristic of DPBS is that it is free of calcium and magnesium ions. These two divalent cations are closely related to cell adhesion on solid surfaces and the cell–cell interaction [29–32].

Without calcium and magnesium ions, we suspected that fewer monocytes would be attached to the surface of the culture flask. Although most of the composition is not revealed in the datasheet provided, the X-VIVO15 medium we used for the study and the clinical trials mentioned in this article contains human native transferrin. One study published in 2019 has shown increased phagocytic capacity and expansion rate of microglial cells treated with transferrin [33]. Microglial cells are mononuclear myeloid cells that reside in the central nervous system (CNS) parenchyma and are involved in immune activity in CNS. Furthermore, it was suggested that dimer and tetramer transferrin was the precursor of glycoproteins released by platelets to activate immune cell phagocytosis [34]. Since many serum-free media for T-cell culture contain human serum proteins, including albumin, transferrin, and fibronectin, to support cell growth and inorganic salt providing calcium ions to regulate membrane potential, it is possible that these media will not be optimal for T-cell isolation. However, more experiments should be done to define the precise mechanisms underlining the observation that monocytes engulfed Dynabeads during incubation in the presence of cell culture media.

In our study, unlike CAR-T cell expansion, CAR transduction efficiency was not affected by the proportion of CD14^+^ cells in PBMC nor by the types of T cell isolation buffer. However, Wang et al. demonstrated that depleting CD14^+^ cells by plastic adhesion could elevate CAR transduction efficiency and CAR-T cell expansion at the same time [26]. One of the reasons behind this discrepancy could be the time point when the CAR-T product was sampled for the CAR transduction efficiency test. CAR transduction efficiency is often examined by flow cytometry detecting the percentage of CAR scFv positive cells in total cells. Since CAR-T cells continue to proliferate after viral transduction, the later the product is sampled, the more dominant CAR scFv positive cells are in the culture and the smaller the gap between groups of different treatments. In the study of Wang et al. they saw the elevation of CAR transduction efficiency in the group treated by plastic adhesion compared to the non-treated in the small-scale CAR-T production, which did not specify the sampling time. There was no non-treated control group in the large-scale production due to the cost of one production run, although they did mention that the test was done on day 11. The other reason is the difference in methodology. In our study, CD14^+^ cells were not depleted, but the T cell isolation buffer change mitigated their capability of inhibiting T cell activation and proliferation. While in the research done by Wang et al. apheresis composition was changed after plastic adhesion.

Noaks et al*.* addressed the problem of the CD14 + cells by enriching apheresis through CD14^+^ cells depletion or using a depletion kit with a combination of blood cell capturing beads other than CD3^+^ T cells [27]. Their study showed no significant difference in CAR transduction efficiency between CD14^+^ cells depletion apheresis and isolated CD3^+^ T cells. Stroncek et al*.* used plastic adherence to deplete CD14 + cells and measured the CAR transduction efficiency for GD2 CAR-T production but not for the CD19 CAR-T production [24, 28]. To conclude, the elevation of CAR transduction efficiency was not a necessary outcome of CD14^+^ cell depletion. It might depend on the methods and CAR-T constructs used in CAR-T production.

There are many methods to deplete CD14^+^ monocytes, including Percoll density gradient centrifugation, plastic adhesion exploiting the characteristic of monocytes, and immunomagnetic beads selection pursuing higher purity with anti-CD14 beads. However, these techniques, used in an academic setting or clinical trials, pose a significant hurdle to developing a cGMP-compliant process suitable for the pharmaceutical manufacture of CAR-T cells. Some methods, such as plate adhesion, are easy to perform in open procedures with culture well-plates and centrifuge tubes; it could often take an unsurmountable effort to adapt this step to a closed system. Removing CD14^+^ monocyte remains an open process, despite all other actions being performed in a closed system [35].

A 9% failure rate has been reported in one of the Tecartus clinical trials [12]. A universal reason for the failures is hard to find because PBMC collected from individual patients exhibit variations. The loss was often attributed to the poor quality of starting materials due to either disease progression and damage of lymphocytes or changes in immune cell composition caused by prior chemotherapy or other treatments. Here we began to address this critical issue by reviewing and analyzing data collected from ongoing clinical trials to uncover the most influential process parameters and performed well-designed experiments to determine the required parameter. We documented that CD14^+^ monocyte retention in the T-cell isolation process results in poor T-cell activation and diminished CAR-T proliferation. We also demonstrated that a simple change of T cell isolation buffer from X-VIVO to DPBS in the CD3/28 Dynabeads co-incubation step dramatically improved the success rate of manufacturing CAR-T products from PBMC with a high level of CD14^+^ monocytes and a low percentage of CD3^+^ T cells. This simple amendment is currently adapted by our ongoing phase I/II clinical trials, and we are planning to use this modification in closed or automated CAR-T manufacturing systems. The simple solution described in this study is the most effective method to eliminate monocyte problems and is readily employed in a close process.

Furthermore, we have successfully modified our manufacturing process. Our ongoing clinical trials showed that CAR-T products manufactured with this new process are highly effective for our MM clinical trial patients. We have improved the manufacturing process with a straightforward change in the T-cell selection buffer. Our study provides an example of how crucial continuous manufacturing process optimization is via discovering manufacturing obstacles in previous clinical trials and doing experiments with the DoE tool.

## Materials and methods

### PBMC Enrichment

PBMC was isolated from apheresis products obtained from consented patients enrolled in clinical trials conducted by Hrain Biotechnology Co., Ltd. The isolation of PBMC fraction was performed with Sepax C-Pro Cell Processing System (Cytiva, Washington D.C.) and its built-in NeatCell C-Pro Protocol developed by the manufacturer. In brief, the tubing system of the bags containing apheresis product, Ficoll (Tianjin Haoyang Biological Manufacture Co., Ltd., Tianjin, China), wash buffer, and freezing media was connected to CT-90.1 C-Pro Sepax Kit (Cytiva, Washington D.C.), which was then loaded onto C-Pro. By running the NeatCell program on the instrument, leukapheresis was fractioned. The PBMC layer was pushed into freezing bags after a few wash rounds and resuspended in freezing media. Freezing bags with PBMC were sealed and separated from the tubing system. PBMC were cryopreserved using a controlled-rate freezer and stored in a liquid nitrogen tank.

### ***CD3***^+^***T cell isolation and activation***

Cryopreserved PBMC was thawed at a 37 ℃-water bath and washed and resuspended in CAR-T culturing medium composed of 1% GlutaMAX (ThermoFisher Scientific, Waltham, MA), 1% HEPES (ThermoFisher Scientific, Waltham, MA), 0.2% N-Acetyl-l-cysteine (CHENG YI, Wenzhou, China) and 5% human plasma in X-VIVO15 (LONZA, Valais, Switzerland). Then, PBMC was rested for 20–24 h at 37 ℃ with 5% CO_2_ in a cell culture flask. Finally, PBMC has been washed and resuspended in an isolation buffer for CD3^+^ T cell selection. This study tested three isolation media: DPBS (Corning Incorporated, Corning, NY), X-VIVO15 (LONZA, Valais, Switzerland) basic medium, and CAR-T culturing medium. X-VIVO15 basic medium consists of X-VIVO15 (LONZA, Valais, Switzerland) with 0.2% NAC (CHENG YI, Wenzhou, China), 1% HEPES (ThermoFisher Scientific, Waltham, MA), and 1% GlutaMAX (ThermoFisher Scientific, Waltham, MA). Positive selection of CD3^+^ T cells was made by co-incubating CTS™ Dynabeads™ CD3/CD28 (ThermoFisher Scientific, Waltham, MA) with PBMC at a 1:1 ratio at room temperature followed by magnetic capture of bead-bound cells on DynaMag™-5 Magnet (ThermoFisher Scientific, Waltham, MA). Selected cells were resuspended in CAR-T culturing medium supplemented with 300 IU/mL IL-2 (SL Pharm, Beijing, China) and incubated at 37 ℃ with 5% CO_2_ for 44–52 h to complete T cell activation.

### Retroviral transduction and CAR T cell proliferation

Before retroviral transduction, the mixture of activated T cells and beads was first applied to a DynaMag™-5 Magnet for the bead removal and then washed with CAR-T culturing medium. Next, gamma retroviral vector carrying CD19 CAR sequence and activated cell suspension were transferred into RetroNectin-coated PermaLife cell culture bags PL70-2G (OriGen Biomedical, Austin, TX) with the addition of 300 IU/mL IL-2 (SL Pharm, Beijing, China). After 18–24 h coculture at 37 ℃ with 5% CO_2_, cells were washed and transferred to a new PL70-2G (OriGen Biomedical, Austin, TX) bag for CAR-T proliferation under the same media and culture condition as during transduction. Again, CAR-T cell numbers were counted, and the cell density was adjusted to 3 × 10^5^ cells/mL every 1–3 days.

### Flow cytometry

Flow cytometry was performed with MACS Quant with the following antibodies: human BD Fc Block (BD Biosciences, Franklin Lakes, New Jersey), Viability Dye (eBioscience, San Diego, CA), Pacific Blue anti-human CD14 Antibody (BioLegend, San Diego, CA), FITC anti-human CD19 Antibody (BioLegend, San Diego, CA), PE-Cy7 anti-human CD56 Antibody (BioLegend, San Diego, CA), APC-Cy7 anti-human CD3 Antibody (BioLegend, San Diego, CA), APC anti-human CD16 Antibody (BioLegend, San Diego, CA), BV421 anti-human CD25 Antibody (BioLegend, San Diego, CA), FITC anti-human CD69 Antibody (BioLegend, San Diego, CA), PerCP anti-human CD8a Antibody (BioLegend, San Diego, CA), PE-Cy7 anti-human CD4 Antibody (BioLegend, San Diego, CA), Biotin Rabbit Anti-Mouse FMC63 scFv (BioSwan, Shanghai, China), and BV421-Streptavidin (BioLegend, San Diego, CA).

### Statistical analysis

GraphPad Prism and JMP were used for conducting statistical analysis.

## Supplementary Information

**Additional file 1: **Cell density in cell-bead co-incubation is not critical in the T cell selection process. Description: During the cell-bead co-incubation step, cell density (ranging from 4*10^6^ to 10*10^6^ cells/mL) did not affect T cell activation, CAR-T proliferation, and CAR transduction efficiency. (a) CD25 expression percentage in CD3^+^ cells, (b) CD69 expression percentage in CD3^+^ cells, and (c) live cell diameter before CD3^+^ T cell selection and after 48-hour T cell activation. In (a), (b), and (c), an unpaired t-test was used, and two-tailed P value was calculated between 4*10^6^ cells/mL and 7*10^6^ cells/mL, 4*10^6^ cells/mL and 10*10^6^ cells/mL, and 7*10^6^ cells/mL and 10*10^6^ cells/mL; ns, P>0.3000. (d) Changes of CD25 expression percentage in CD3^+^ cells, (e) CD69 expression percentage in CD3^+^ cells, and (f) live cell diameter in the CAR-T manufacturing process. (g) Expansion fold of CAR-T cells after retroviral transduction. (h) CAR expression percentage in CD3^+^ cells at days 6 and 9 after retroviral transduction.

**Additional file 2: **Cell-bead co-incubation duration is not critical in the T-cell selection process. The cell-bead co-incubation time, ranging from 30 minutes to 60 minutes, during T cell selection did not affect T cell activation, CAR-T proliferation, and CAR transduction efficiency. (a) CD25 expression percentage in CD3^+^ cells, (b) CD69 expression percentage in CD3^+^ cells, and (c) live cell diameter before CD3^+^ T cell selection and after 48-hour T cell activation. In (a), (b), and (c), an unpaired t-test was used, and a two-tailed P value was calculated between 30 minutes and 45 minutes, 30 minutes and 60 minutes, and 45 minutes and 60 minutes; ns, P>0.3000. (d) Changes in CD25 expression percentage in CD3^+^ cells, (e) CD69 expression percentage in CD3^+^ cells, and (f) live cell diameter in the CAR-T manufacturing process. (g) Expansion fold of CAR-T cells after retroviral transduction. (h) CAR expression percentage in CD3^+^ cells at days 6 and 9 after retroviral transduction.

## Data Availability

The datasets generated and analyzed during the current study are not publicly available due to the confidentiality agreement of Hrain Biotechnology Co., Ltd. Still, they are available from the corresponding author on reasonable request.

## Abbreviations

ALL: Acute lymphoblastic leukemia
BCMA: B-cell maturation antigen
CAR: Chimeric antigen receptor
CNS: Central nervous system
CRS: Cytokine release syndrome
DoE: Design of experiment
DPBS: Dulbecco’s phosphate-buffered saline
FDA: U.S. Food and Drug Administration
GM-CSF: Granulocyte–macrophage colony-stimulating factor
ICONS: Immune effector cell-associated neurotoxicity syndrome
IIT: Investigator-initiated trials
IL-2: Interleukin 2
MM: Multiple myeloma
NHL: Non-Hodgkin’s lymphoma
PBMC: Peripheral blood mononuclear cells
PSC: Peripheral stem cells
