# Supplementary figures and images for: A simple and effective method to purify and activate T cells for successful generation of chimeric antigen receptor T (CAR-T) cells from patients with high monocyte count
Source: J Transl Med. 2022 Dec 19;20:608. doi: 10.1186/s12967-022-03833-6 (PMC9764707; doi:10.1186/s12967-022-03833-6)

**a**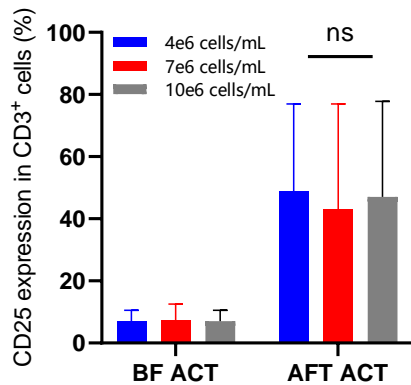**b**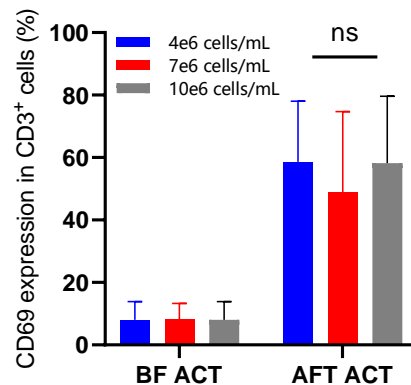**c**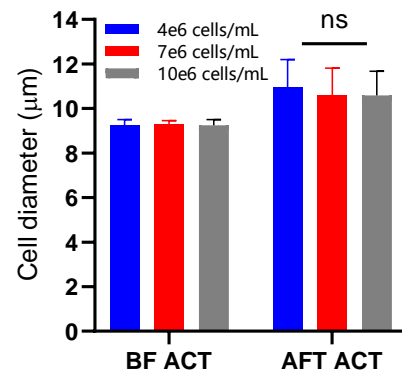**d**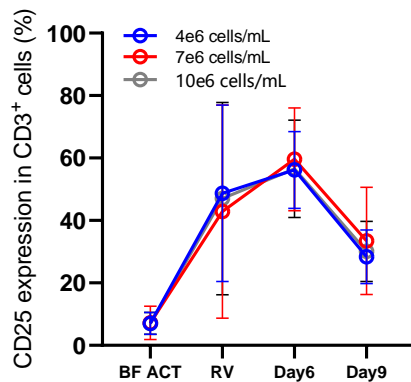**e**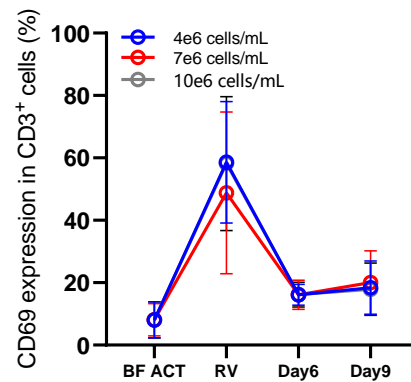**f**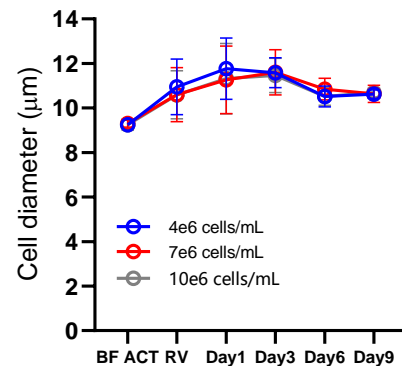**g**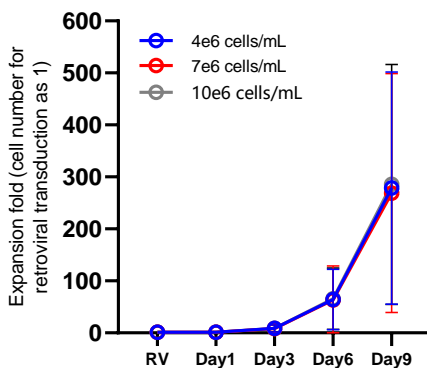**h**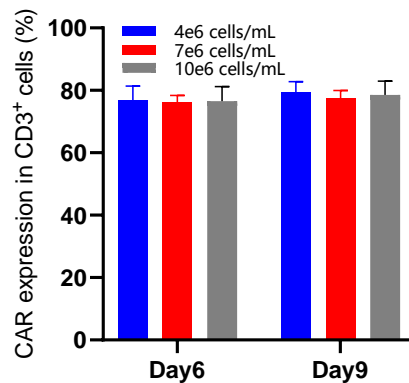

Supplement: Supplementary file 1 — Additional file 1: Cell density in cell-bead co-incubation is not critical in the T cell selection process. Description: During the cell-bead co-incubation step, cell density (ranging from 4*106 to 10*106 cells/mL) did not affect T cell activation, CAR-T proliferation, and CAR transduction efficiency. (a) CD25 expression percentage in CD3+ cells, (b) CD69 expression percentage in CD3+ cells, and (c) live cell diameter before CD3+ T cell selection and after 48-hour T cell activation. In (a), (b), and (c), an unpaired t-test was used, and two-tailed P value was calculated between 4*106 cells/mL and 7*106 cells/mL, 4*106 cells/mL and 10*106 cells/mL, and 7*106 cells/mL and 10*106 cells/mL; ns, P>0.3000. (d) Changes of CD25 expression percentage in CD3+ cells, (e) CD69 expression percentage in CD3+ cells, and (f) live cell diameter in the CAR-T manufacturing process. (g) Expansion fold of CAR-T cells after retroviral transduction. (h) CAR expression percentage in CD3+ cells at days 6 and 9 after retroviral transduction. [file 12967_2022_3833_MOESM1_ESM.pdf]

**a**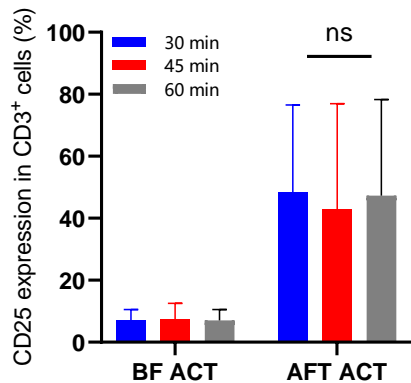**b**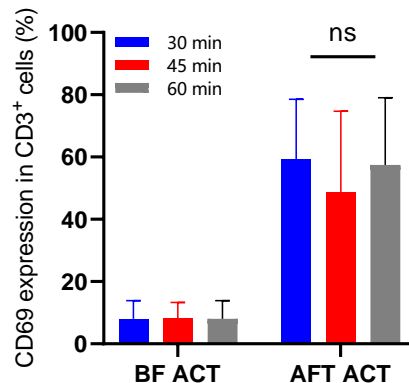**c**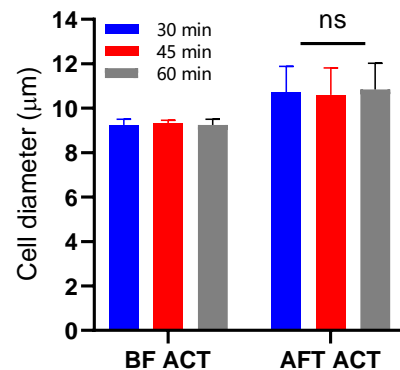**d**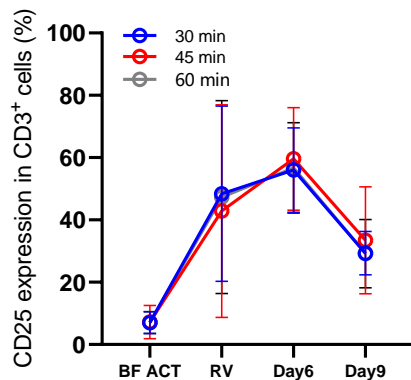**e**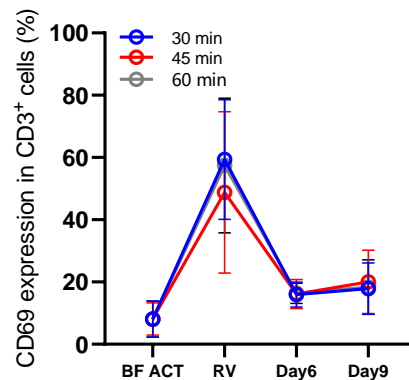**f**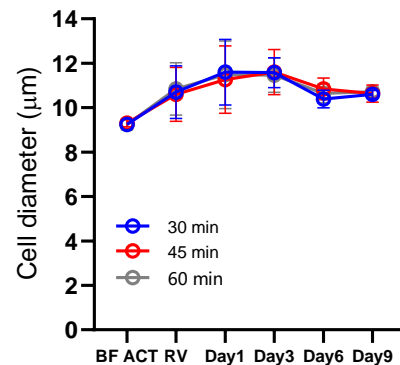**g**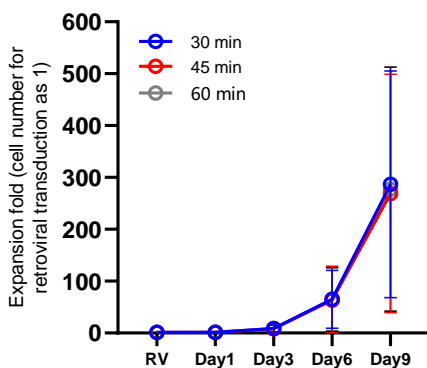**h**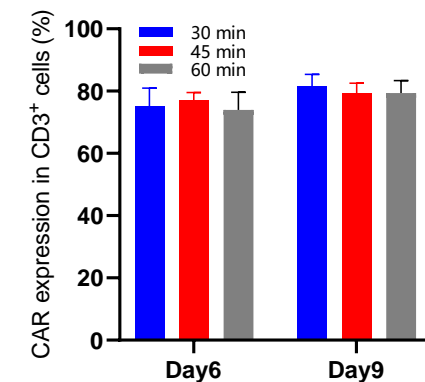

Supplement: Supplementary file 2 — Additional file 2: Cell-bead co-incubation duration is not critical in the T-cell selection process. The cell-bead co-incubation time, ranging from 30 minutes to 60 minutes, during T cell selection did not affect T cell activation, CAR-T proliferation, and CAR transduction efficiency. (a) CD25 expression percentage in CD3+ cells, (b) CD69 expression percentage in CD3+ cells, and (c) live cell diameter before CD3+ T cell selection and after 48-hour T cell activation. In (a), (b), and (c), an unpaired t-test was used, and a two-tailed P value was calculated between 30 minutes and 45 minutes, 30 minutes and 60 minutes, and 45 minutes and 60 minutes; ns, P>0.3000. (d) Changes in CD25 expression percentage in CD3+ cells, (e) CD69 expression percentage in CD3+ cells, and (f) live cell diameter in the CAR-T manufacturing process. (g) Expansion fold of CAR-T cells after retroviral transduction. (h) CAR expression percentage in CD3+ cells at days 6 and 9 after retroviral transduction. [file 12967_2022_3833_MOESM2_ESM.pdf]
